# A Molecularly Characterized Preclinical Platform of Subcutaneous Renal Cell Carcinoma (RCC) Patient-Derived Xenograft Models to Evaluate Novel Treatment Strategies

**DOI:** 10.3389/fonc.2022.889789

**Published:** 2022-06-21

**Authors:** Dennis Gürgen, Michael Becker, Mathias Dahlmann, Susanne Flechsig, Elke Schaeffeler, Florian A. Büttner, Christian Schmees, Regina Bohnert, Jens Bedke, Matthias Schwab, Johann J. Wendler, Martin Schostak, Burkhard Jandrig, Wolfgang Walther, Jens Hoffmann

**Affiliations:** ^1^ Experimental Pharmacology and Oncology Berlin-Buch GmbH (EPO), Berlin, Germany; ^2^ Dr. Margarete Fischer-Bosch Institute of Clinical Pharmacology, Stuttgart, Germany; ^3^ Cluster of Excellence iFIT (EXC 2180) “Image-Guided and Functionally Instructed Tumor Therapies”, University of Tübingen, Tübingen, Germany; ^4^ German Cancer Consortium (DKTK), Partner Site Tübingen, German Cancer Research Center (DKFZ), Heidelberg, Germany; ^5^ Natural and Medical Sciences Institute (NMI) at the University of Tübingen, Reutlingen, Germany; ^6^ Department of Urology, University Hospital Tübingen, Tübingen, Germany; ^7^ Departments of Clinical Pharmacology, and Pharmacy and Biochemistry, University of Tübingen, Tübingen, Germany; ^8^ Department of Urology, University Medical Center Magdeburg, Magdeburg, Germany; ^9^ Experimental and Clinical Research Center (ECRC) Charité Universitätsmedizin Berlin, Berlin, Germany; ^10^ Max-Delbrück-Centrum für Molekulare Medizin (MDC), Berlin, Germany

**Keywords:** kidney cancer, renal cell carcinoma (RCC), clear cell RCC, patient-derived xenograft (PDX), preclinical oncology, targeted therapy, immuno-oncology

## Abstract

Renal cell carcinoma (RCC) is a kidney cancer with an onset mainly during the sixth or seventh decade of the patient’s life. Patients with advanced, metastasized RCC have a poor prognosis. The majority of patients develop treatment resistance towards Standard of Care (SoC) drugs within months. Tyrosine kinase inhibitors (TKIs) are the backbone of first-line therapy and have been partnered with an immune checkpoint inhibitor (ICI) recently. Despite the most recent progress, the development of novel therapies targeting acquired TKI resistance mechanisms in advanced and metastatic RCC remains a high medical need. Preclinical models with high translational relevance can significantly support the development of novel personalized therapies. It has been demonstrated that patient-derived xenograft (PDX) models represent an essential tool for the preclinical evaluation of novel targeted therapies and their combinations. In the present project, we established and molecularly characterized a comprehensive panel of subcutaneous RCC PDX models with well-conserved molecular and pathological features over multiple passages. Drug screening towards four SoC drugs targeting the vascular endothelial growth factor (VEGF) and PI3K/mTOR pathway revealed individual and heterogeneous response profiles in those models, very similar to observations in patients. As unique features, our cohort includes PDX models from metastatic disease and multi-tumor regions from one patient, allowing extended studies on intra-tumor heterogeneity (ITH). The PDX models are further used as basis for developing corresponding *in vitro* cell culture models enabling advanced high-throughput drug screening in a personalized context. PDX models were subjected to next-generation sequencing (NGS). Characterization of cancer-relevant features including driver mutations or cellular processes was performed using mutational and gene expression data in order to identify potential biomarker or treatment targets in RCC. In summary, we report a newly established and molecularly characterized panel of RCC PDX models with high relevance for translational preclinical research.

## Introduction

Renal cell carcinoma (RCC) and clear cell renal cell carcinoma (ccRCC) as its major subtype represent 80% of all RCC malignancies and belong to the 10 most commonly diagnosed cancers. The prevalence of RCC in adults is higher in men than in women, with a ratio of 1.6–1.9:1.0. This disease mainly occurs during the sixth or seventh decade of patient lives, with an average onset at 64 years (1). In addition to ccRCC, RCC comprises a variety of subtypes as outlined in the 2016 World Health Organization (WHO) classification (2). Less common kidney cancer subtypes include papillary (10%–15%), chromophobe (5%), and other rare tumors of the nephron and collecting duct system. Of note, these subtypes arise from different parts of the nephron. Whereas the main subtypes ccRCC and papillary RCC arise from proximal tubules, chromophobe RCC is supposed to originate from distal parts of the nephron (3, 4). In ccRCC, the *VHL* gene, mapped on human chromosome 3p25, has been implicated in both the hereditary and sporadic forms. Under physiological conditions, the VHL protein functions as a tumor suppressor (5). Heterozygous inheritance of the *VHL* allele results in a high risk of developing ccRCC (6). Further frequent somatic gene mutations in familial RCC had been discovered including *BAP1, CDKN2A, MET, PBRM1, PTEN, SETD2, TP53*, and *TSC1*, some of which also occur in sporadic forms of the disease and correlate with decreased survival (3).

Advanced imaging techniques enable early detection of localized pT1a RCC, providing the best opportunity for complete tumor dissection. Hence, the 5-year survival rate of pT1a tumors is approximately 95%, which rapidly declines with higher stage, grade, and metastasis. VEGF receptor tyrosine kinase inhibitors (RTKIs) have been the first targeted drugs introduced for the treatment of advanced RCC (7, 8). A second class of small molecules successfully utilized for the treatment in RCC include mTOR inhibitors (9, 10). Recently, the combination of ICIs targeting the PD-1/PD-L1 axis with VEGF RTKI demonstrated superior efficacy in a randomized phase III trial (11). The combination of cabozantinib and nivolumab improved the clinical outcome in treatment-naive advanced RCC compared to monotherapy with sunitinib with a median progression-free survival of 16.1 vs. 8.3 months (12). Consequently, the combination of immune checkpoint blockade and VEGF inhibition rapidly evolved as a novel standard in first-line therapy of RCC and is recommended by guidelines (13).

RCC is a heterogeneous tumor, and despite highly successful targeted therapies in primary RCC, treatment of advanced metastatic disease remains a therapeutic challenge. A leading determinant of treatment resistance in RCC might be a result of pronounced intra-tumor heterogeneity (ITH). Factors contributing to distinct ITH like specific histology, and variable and diverse molecular features account for poor survival in metastatic disease (14). Additionally, adaptive and acquired drug resistance is frequently observed in a high proportion of treated patients, and the underlying molecular mechanisms are not yet clearly understood (15).

In comparison to the next-generation VEGF RTKI, the therapeutic effect of mTOR inhibitors, mainly used in the second line (16), was limited. Therefore, the use of mTOR inhibitors might be more reasonable in combination therapies (17). Elevated occurrence of private mutations and expanded tumor heterogeneity in progressed or relapsed metastatic RCC correlate with poor prognosis (18). Additionally, targeting acquired TKI resistance mechanisms in advanced and metastatic RCC remains an unsolved issue, any or all of which require the development of novel therapies for progressed and metastatic RCC to address this high medical need.

PDX models are a validated methodology in preclinical oncology research with high translational relevance (19, 20). It has been reproducibly shown that especially large panels of PDX models can capture unique properties of patient tumors *in vivo* and can serve as a valuable tool for studying disease mechanisms and patient-specific therapy responses (21). With the current study, we aimed to establish a comprehensive panel of molecularly characterized subcutaneous RCC PDX models, which enables both the preclinical evaluation of novel targeted therapies in correlation to clinical response and the identification of new molecular targets. In addition, this RCC PDX platform can be used to identify and validate RCC-specific biomarkers and, if used on humanized mice, to further refine the use of immuno-oncology combination therapies in personalized medicine.

## Materials and Methods

### Human Tumor Tissues

Use of patient tissue was approved by the local ethics committees of the Otto-von-Guericke University Magdeburg (87/11, Magdeburg cohort) and the University of Tübingen (622/2020BO, Tübingen cohort). Patient authorization and informed consent were given before tissue collection and execution of experiments.

Human renal carcinoma tissues were retrieved from clinical surgery and transplanted into mice within 24 h. In total, 218 specimens from primary and metastatic RCCs from 167 consenting patients were collected. The establishment and characterization of PDX models were carried out as previously reported (22, 23). In brief, after removal of transport media (RPMI 1640 and 50 µg/ml Gentamicin, both Gibco™, Thermo Fisher Scientific Inc., Bartlesville, OK, USA), the primary human tumor material was sliced into small pieces, rinsed with PBS (Gibco), and subcutaneously (s.c.) transplanted with matrigel supplemented with growth factors (Corning Matrigel Basement Membrane Matrix, Corning GmbH, Kaiserslautern, Germany) into the left flank of anesthetized mice. Engrafted and growing PDX were allowed to reach a tumor volume (TV) of approximately 1 cm³ before retrieval and consecutive *in vivo* passaging. Xenograft tissue was routinely conserved as vital tissue in FCS/DMSO in liquid nitrogen, snap frozen, and stored at −80°C or prepared as a formalin-fixed, paraffin-embedded (FFPE) specimen.

### Development and Establishment of RCC Patient-Derived Xenograft Models

The work conducted in animals is in accordance to the German Animal Welfare Act, and all procedures were approved by local authorities (Landesamt für Gesundheit und Soziales, LaGeSo Berlin, Germany) under approval number H0032-09 for *in vivo* PDX passages and A0452-08 for preclinical sensitivity experiments. All mice were handled in accordance with the Guidelines for the Welfare and Use of Animals in Cancer Research (24). We do not have evidence for a particular bias of the gender from passage mice for the success rate of RCC PDX establishment. In general, and if possible, the gender of mice for the primary *in vivo* s.c. transplantation is identical to the gender of the patient from which the tumor derived. Animals were IVC housed under sterile and standardized conditions (22°C ± 1°C, 50% relative humidity, 12-h light–dark cycle, autoclaved food, bedding material, and tap water *ad libitum*).

### Histology and Immunohistochemistry

FFPE blocks were sectioned (5 μm), deparaffinized, and rehydrated. Specimens were stained according to standard hematoxylin–eosin protocol for histopathological evaluation. For IHC staining, 3-µm-thick serial sections of RCC PDX tumor specimen were cut from FFPE tissue samples. Antigen retrieval for staining of *CD31*, *Pax2*, and *Pax8* was performed in a steamer for 20 min in citrate buffer, pH 6.0. Unspecific binding epitopes were blocked with 4% BSA in PBS for 1 h at room temperature (RT). Tissue incubation with primary antibodies—*Ki-67* polyclonal rabbit anti-human *Ki-67* antigen (Abcam #ab15580, Germany), *CD31* polyclonal rabbit anti-human (Abcam #ab28364, Germany), and rabbit anti-human *Pax2* and *Pax8* (#PI593C002 and #PI924C002, DCS, Germany)—was conducted 45 min at RT. Detection of *Pax2* and *Pax8* primary antibodies was performed with EnVision-mouse HRP (Dako, Germany) for 45 min at RT. For detection and visualization, the Signal Stain Boost IHC Detection reagent (#8114S, Cell Signaling Technology, Germany) or Super Vision RED 2 kit (#AD000POL-K, DCS, Germany) was used. After several washes in PBS, sections were stained in Mayer’s hemalum (Carl Roth, Karlsruhe, Germany) for 2 min. After washing in water for 10 min, cover slips were fixed with Dako coverslipper using Dako mounting medium (Dako, Denmark). Validation of IHC staining specificity including the use of negative controls without primary antibody incubation was carried out before the application. Representative pictures from IHC stainings were taken using the Axioskop 40 (100-fold magnification and AxioVision 4.5 the software, both Zeiss, Germany).

### *In Vivo* Drug Response Testing of RCC PDX Models

Thirty-five PDX models of the established RCC cohort of forty-six PDX models were analyzed for their response Standard of Care (SoC) compounds, with ongoing response evaluation for recently established PDX models. Tumor fragments of 3 × 3 mm were subcutaneously transplanted to individual study cohorts utilizing female Rj:NMRI-Foxn1^nu/nu^ nude (Janvier Labs, France) or NOD.Cg-Prkdc^scid^ Il2rg^tm1Sug^/JicTac (Taconic, Denmark) recipient mice. A digital caliper was used to measure palpable tumor dimensions in width [mm] and length [mm], always reporting the smaller diameter as width and the perpendicular diameter as length. Individual TVs were calculated by the equation:


$$\text{TV }{\text{[cm}}^{3}]\text{ = }\frac{\text{length}\times {\text{width}}^{2}}{2}$$

The relative TV of individual tumors on a specific day “x” (RTVx) was calculated using the absolute TV [mm^3^] of respective tumors on day “x” (dx) and the absolute TV of the same tumor on the day of randomization day 0 (d_0_) by the equation:


$$\text{RTV}\times \left[\%\right]=\frac{\text{TVx}}{\text{TVo}}\;\times \;100$$

Once the tumors reached the predefined mean starting volume (0.1–0.2 cm³), mice were randomly assigned to control and treatment groups. Mice were treated with intraperitoneal injection for bevacizumab 10 mg/kg (Avastin, Roche Pharma AG, Basel, Switzerland) and orally with everolimus 5 mg/kg (Certican, Novartis Pharma GmbH, Nuremberg, Germany), sorafenib 80 mg/kg (Nexavar, Bayer Pharma AG, Berlin, Germany), or sunitinib 50 mg/kg (Sutent, Pfizer Pharma GmbH, Berlin, Germany) for 5 consecutive days on treatment and 2 days off treatment (5 on/2 off) for variable cycles depending on the actual tumor growth of the control group. SoC concentrations were selected based on their clinical application and an allometric scaling approach was used to extrapolate doses of the respective drugs among species (25). Based on previously conducted treatment response characterization experiments, the selected drug concentrations indicate good tolerability and preclinical efficacy. For evaluation of therapeutic response, the ratio of the mean TV of the treated group (T) and the control group (C) was expressed as the T/C value in percentage. The anti-tumor activity of the tested compounds in RCC PDX models was classified using the adopted clinical response criteria for solid tumors (RECIST). We considered a T/C >50% = resistance/progressive disease (PD), > 30% ≤ 50% = minor response/stable disease (SD), >5% ≤ 30% = moderate response/partial response (PR), < 5% = strong response/complete remission (CR) (**Figure 4A**). Relative tumor volume (RTV) was calculated as the ratio of the TV on the last day before the end of the study and the TV on the first day of treatment. We defined RTV < 1.6 = strong response, 1.6–2.5 = good response, 2.5–5.5 = minor response, RTV > 5.5 = resistance.

Health status and body weight (BW) for all mice were recorded on a regular basis (data not shown), at least twice weekly in order to control for toxic adverse effects.

### Transcriptome Sequencing, Data Processing, and Data Analysis

RNA sequencing (RNASeq) of one tumor tissue sample each was performed for twenty-eight established RCC PDX models, comprising 17 clear cell, 8 papillary RCC, and 3 urothelial carcinomas. Next-generation sequencing and bioinformatic raw data processing were performed by ATLAS Biolabs GmbH (Berlin, Germany).

### Total RNA Isolation and Sequencing

Snap-frozen PDX tumor tissue (50–100 mg) was disrupted in 1.5 ml of TRIzol™ (ThermoFisher Scientific, Germany) using a gentle MACS dissociator and M tubes (Miltenyi Biotec, Germany) and total RNA was isolated. The RNA integrity was evaluated with the Agilent Bioanalyzer 2100 and the RNA 6000 Nano Kit (Agilent, Germany). The Illumina TrueSeq Stranded mRNA Library Prep Kit was used for preparation of RNASeq libraries following a 100-bp PE-sequencing run on an Illumina HiSeq 2500 device with a depth of 80–100 million reads (40–50 Mio cluster) (Illumina, Cambridge, UK).

### Data Processing

The quality of transcript reads was validated with FastQC (26) version 0.11.8. Xenome (27) version 1.0.1 was used for the classification of xenograft-derived sequence reads (human/mouse read splitting) and referenced to human genome hg38 as graft reference as well as mouse genome mm10 as host reference. STAR aligner (28) version 2.6.1a was used to map human specific reads against the *Homo sapiens* reference hg38. Quality of mapping was validated with QualiMap (29) version 2.2.1 and quantification of transcripts was performed by eXpress (30) version 1.5.1.

### Analysis of Copy Number Variations

Copy number variations (CNVs) on the chromosome-arm level were predicted from quantified gene expression and mutational analysis using RNAseqCNV (https://github.com/honzee/RNAseqCNV).

### Gene Expression Analysis

RNAseq raw count data were transformed to gene length corrected trimmed mean of M-values (GeTMM) (31) to perform single-sample gene set enrichment analyses (ssGSEAs) (32) (https://github.com/broadinstitute/ssGSEA2.0) regarding cancer hallmarks and angiogenesis/hypoxia-related reactome pathways (33). Gene sets to indicate the angiogenic or T-effector tumor type were generated with a recently published 66-gene signature for RCC (34).

### Mutational Analysis in PDX Models

A high sequencing depth of 80–100 million reads within our RNASeq allowed sensitive mutational analysis of the transcriptome including the detection of fusion events. Variant calling and annotation of mapped reads were conducted with GATK 4.0.2.1 (https://github.com/broadinstitute/gatk/releases) (35) and the Ensemble Variant Effect Predictor (VEP), release 94 (https://www.ensembl.org/info/docs/tools/vep/index.html) (36). The subsequent mutational analysis was performed for a set of 31 RCC-relevant genes (37). In order to identify somatic alterations, quality-tested variant calls were filtered based on allele frequencies from gnomAD 3.1. Only variants that either were not included in gnomAD 3.1 or had a gnomAD allele frequency below 0.0001 were considered (**Supplementary Table S1**).

### NGS Panel Sequencing in Corresponding Primary Tumors and Metastases

NGS panel sequencing of primary tumor and metastasis tissue was performed as previously described (37). Briefly, the panel includes 32 genes that are known to play an important role in RCC development and progression. Due to known alignment/sequencing problems, *MUC4* was excluded from variant detection. The detected variants were filtered based on allele frequencies from gnomAD 3.1 to identify somatic mutations. Only variants that were either not included in gnomAD 3.1 or had a gnomAD allele frequency below 0.0001 were considered. Genomic positions of variants found only in PDX models were manually examined for supporting evidence in the panel sequencing data.

### Transcriptome Analysis in Primary Tumors and Metastases

Transcriptome analysis using GeneChip™ Human Transcriptome Array 2.0 (Thermo Fisher Scientific) was performed as previously described (38) for primary tumors and metastases that were collected for the generation of PDX models and for which sufficient primary or metastatic tissue was available. Calculation of the S3 score was performed as described (37, 39) for primary tumors and metastases that were collected for the generation of PDX models. Classification into S3 score risk groups (high/low) was based on the cutoff value defined in Büttner et al. (39).

## Results

The aim of the current study was to establish an RCC PDX platform for preclinical applications including biomarker validation, *in vivo* response characterization, and testing of novel targeted therapies.

**Figure 1:**
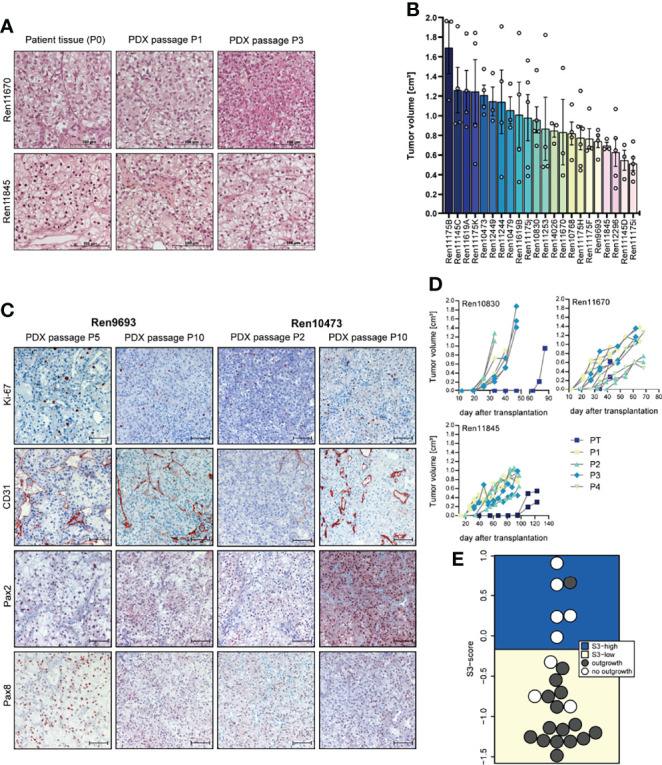
Histopathology, tumor growth characterization, and immunostainings (IHC) of representative PDX models from the RCC PDX panel. **(A)** Histological examination of patient and PDX tissue from the first (P1) and third (P3) PDX *in vivo* passage. FFPE tissue was used for 5-µm sections and standard H&E stainings. **(B)** Tumor growth characteristic of untreated control mice reflecting the heterogeneous biology of RCC regardless of the molecular phenotype. Data from twenty-two RCC PDX models utilized for drug testing studies as mean TV ± SEM, *n* = 3–6. **(C)** Representative IHC analyses from RCC *in vivo* passaged PDX tumors for *Ki-67* (proliferation), CD31 or *PECAM1* (blood vessels), and *Pax2* and *Pax8* (renal marker). The brown staining indicates the positivity for the respective markers within the tissue. Scale bar = 100 µm. **(D)** Exemplary RCC PDX growth curves showing individual TV growth characteristic during *in vivo* passaging (PT = primary tumor passage, P1–P4 indicate consecutive PDX tumor passages). **(E)** The gene expression-based ccRCC risk model (S3 score) was calculated for primary tumors and metastases from tissue of the Tübingen cohort collected for PDX generation. In 16 of the 24 cases shown, the PDX establishment was successful. The risk categories of the S3 score are represented by the background color. A high S3 score is equivalent to a good prognosis, while a low S3 score corresponds to a poor prognosis in terms of cancer-specific survival, indicating a correlation between S3 score and successful PDX establishment.

After receiving 218 primary biopsies from 167 patients suffering from renal cancer, a panel of 46 PDX models was established *via* subcutaneous transplantation of vital tumor tissue (**Table 1**). This panel includes 33 ccRCC PDX models from 29 patients, 8 papillary RCC PDX models from primary tumor and its respective lymph node metastasis from one patient, 1 chromophobe and 1 sarcomatoid RCC PDX model, as well as 3 urothelial carcinoma PDX models. The median age of all donors was 61 years (range, 37–86 years). Twenty-seven PDX models originated from male patients (59%), 18 from female patients (39%), and 1 of unknown gender (2%) (**Table 1**). Ten RCC PDX models emerged from metastatic tissue, two from undefined origin, and thirty-four were retrieved from primary and mainly untreated RCC tumor tissues. Clinical response data to SoC or TKI were allocated for seven patients (Ren11145, Ren11619, Ren11244, Ren11254, Ren11324, Ren11670, and Ren12147). Number and time for *in vivo* passage for all established RCC PDX models were recorded, and the PDX tumors were characterized by histopathology and immunohistochemistry. Representative results were illustrated for selected models (**Figures 1A–D**).

**Table 1:** Clinical and pathological characteristics of RCC patients providing primary kidney tumor tissue for the establishment of PDX models.

ID#	Age	Gender	TNM	Grading	Histology	Tumor biopsy
9693	n/a	Male	n/a	n/a	Clear cell RCC	Primary
10473	74	Female	pT4 pN0 M1	G3	Clear cell RCC	Primary
10479	72	Male	pT3a pN0 M0	G3	Clear cell RCC	Primary
10768	63	Male	pT3a pN2 M1	G3	Clear cell RCC	Primary
10830	77	Female	pT1a pN0 M0	n/a	Clear cell RCC	Primary
11122D	45	Female	pT2a pN0 M1	G3	Clear cell RCC	Primary—region 4
11122E						Primary—region 5
11122F						Primary—region 6
11145C	52	Male	pT3a Nx M1	G3-4	Clear cell RCC	Primary—region 3
11145D						Primary—region 4
11175B	60	Male	pT3b pN2 M1	G3	Papillary RCC*	Primary—region 3
11175C						Primary—region 4
11175D						Primary—region 5
11175F						LN** metastasis—region 7
11175H						LN** metastasis—region 9
11175i						LN** metastasis—region 10
11175J						LN** metastasis—region 11
11175K						LN** metastasis—region 12
11201	86	Female	pT3a pN1 L1	G3	Urothelial carcinoma	Primary
11244	69	Female	pT3a NX M1	G2	Clear cell RCC	Primary
11253	71	Male	pT2a pN0 M0	G2	Clear cell RCC	peritoneal metastasis
11254	61	Female	pT3a pN2 L1	G3	Clear cell RCC	Primary
11324D	75	Female	pT3a Nx M0	G3	Clear cell RCC	Primary—region 4
11325H	64	Female	pT4 Nx M0	G3	Clear cell RCC	Primary—region 8
11535	83	Male	n/a	n/a	Clear cell RCC	Primary
11619A	55	Male	pT3a NX L1	G3	Clear cell RCC	Primary—region 1
11619B						Primary—region 2
11644	63	Male	pT1b pNX cM1	G2	Clear cell RCC	Bone metastasis
11670	83	Female	pT2a pN0 M1	G2	Clear cell RCC	Bone metastasis
11845	83	Female	pT2a R0 L0 V0	G2	Clear cell RCC	Primary
11965	63	Male	n/a	n/a	Clear cell RCC	n/a
12147	78	Male	pT4 pN1 M1	G3	Urothelial carcinoma	Primary
12296	50	Male	pT3a, R0, L0, V2, Pn0	G4	Clear cell RCC	Primary
12449	37	Male	pT3b pN2 M1	G3	Urothelial carcinoma	Spine metastasis
12522	64	Female	pT1b R0 L0 V0 Pn0	G4	Clear cell RCC	Primary
12723	68	Female	pT3a (m), pN0 (0/2) pM1, R0, L, V0, Pn0	G3	Clear cell RCC	Primary
12739	85	Male	pT1a L0 V0 R0	G1	Clear cell RCC	Primary
12813	46	Female	pT3a L1 V1 R0	G4	Clear cell RCC	Primary
12837	n/a	Male	n/a	n/a	Clear cell RCC	n/a
13311	74	Male	pT3b, pM1 (OTH), V2, L1, R0	G3	Clear cell RCC	Primary
13461	58	Male	pT4 pM1 (OTH) V2 L1 R1	n/a	Sarcomatoid RCC	Primary
13581	76	Female	pT1b V0 L0 R0	G4	Chromophobe RCC	Primary
13622	74	Female	pT3a pN1 (2/2) V2 R0	G3	Clear cell RCC	Primary
14026	n/a	n/a	n/a	n/a	Clear cell RCC	Lung metastasis
14444	57	Male	pT3a L0 V2 G3 R1	G3	Clear cell RCC	Primary
16378	47	Female	pT3a, pM1(ADR) L0 V2 Pn0 R1	G4	Clear cell RCC	Primary

* with in part clear cell renal cell carcinoma features.

** lymph node.

n/a stands for "not available".

The overall take rate for RCC PDX was determined as 21% and is comparable to previously reported engraftment success rates in other RCC PDX studies (40). However, the probability for successful PDX engraftment strongly correlated with the individual tumor stage. A high positive correlation was found with RCC grade. For tumor tissue samples from grades 3 and 4, we noticed a take rate of 85% and 83%, respectively. Furthermore, engraftment of ccRCC correlated with a recently published gene expression-based prognostic score (S3 score) (39) (**Figure 1E**). This score models the positive correlation between survival and gene expression similarity of tumor tissue to the S3 region of proximal tubules, which is the presumed region of ccRCC origin. Consistently, a high S3 score, indicating high gene expression similarity, was strongly associated with failed engraftment after s.c. transplantation into immune-deficient mice (5 out of 6), whereas successful PDX model establishment (15 out of 18) was based on tumors with a low S3 score.

Morphology and histopathology between primary human and respective PDX tumors were well conserved throughout consecutive *in vivo* passaging in mice, and similar patterns of proliferation (*Ki-67*), vascularization (*CD31*), and renal cell marker expression (*Pax2* and *Pax8*) were detected over several PDX passages (**Figures 1A, C**). Each PDX model exhibits its own unique growth characteristic and the tumor growth in control mice during sensitivity testing was highly variable over a broad range, reflecting the heterogeneity of RCC samples in the PDX models (**Figure 1B**). This variability was already noticed during early RCC PDX passaging with large differences in engraftment time of primary tumors. As shown in **Figure 1D**, tumor growth is even highly variable within one xenograft model, illustrating high intra-tumoral differences in the molecular phenotype. We further observe an increase in tumor growth with increasing *in vivo* passage numbers, which might be caused by stepwise selection of more adaptive tumor clones to the altered tumor microenvironment in the immune-deficient mice and the potential loss of suppressive human immune cells in the xenograft.

### Mutational Landscape of Primary Patient Tumors/Metastasis and Renal Cell Carcinoma PDXs

**Figure 2:**
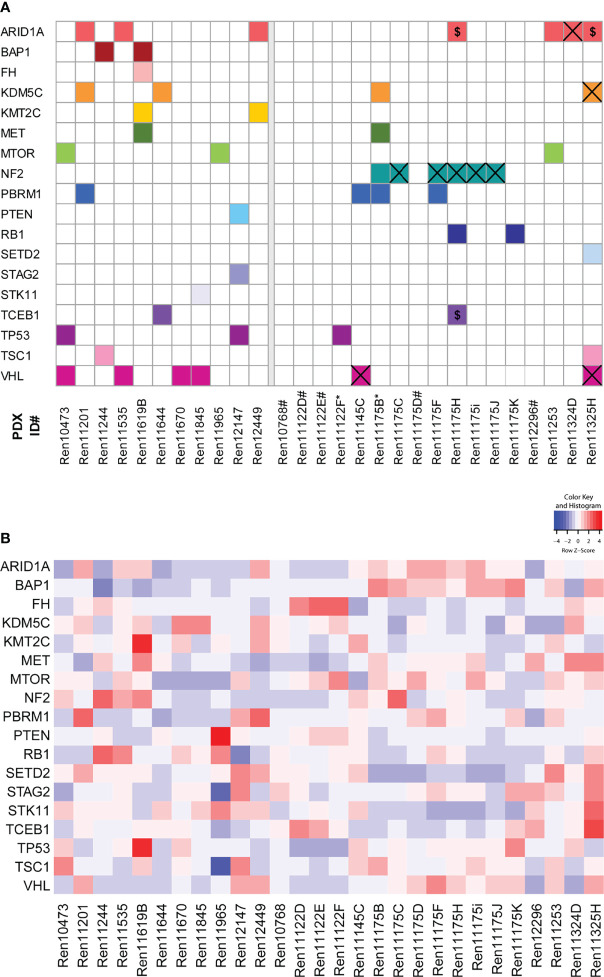
Somatic mutation analysis in RCC PDX models and expression of mutated genes. **(A)** Using RNA sequencing data, 31 genes that are frequently mutated in RCC were analyzed for somatic mutations. The matrix includes those variants in 18 genes either not included in gnomAD 3.1 or had allele frequencies below 0.0001. The panels separate the models by the tissue source site of their primary tumors and metastases (left: Magdeburg, right: Tübingen). For the Tübingen cases, mutation data from NGS panel sequencing of the primary tumors were also available (except for those marked with *). Overlapping mutations detected in both the primary tumor and the PDX model are highlighted by a cross. $: Variants with low coverage in the primary tumors. #: No somatic mutation found. **(B)** Gene expression of the 18 mutated candidate genes described in **(A)**.

Mutational analysis was performed using RNA sequencing data from 28 RCC PDX models and compared to somatic mutation data from panel sequencing for a subset of primary human tumors from the Tübingen cohort (**Figure 2A**, right panel). In the analysis, we focused on 31 key genes that are frequently mutated in RCC (37, 41). Somatic mutations were found in 18 different genes and in 23 PDX models from which 15 PDX models cover more than one gene aberration. The mutations of *NF2* and *VHL* are present in both the PDX model and the corresponding primary tumor in the Tübingen cohort (**Figure 2A** right, crossed squares). When only considering these two mutated genes, the rate of conserved mutations between patient tumor and PDX tissue was very high, resulting in 100% for *NF2* and 100% for *VHL*, respectively. Interestingly, the *NF2* mutation was present in 6 out of 8 PDX models of one patient. Additionally, we used our data to visualize the relative transcript expression of mutated genes in our panel (**Figure 2B**). Hereby, similarities and common features of individual models can be visualized, which enables target specific selection of RCC PDX models for preclinical applications.

### Gene Expression

**Figure 3:**
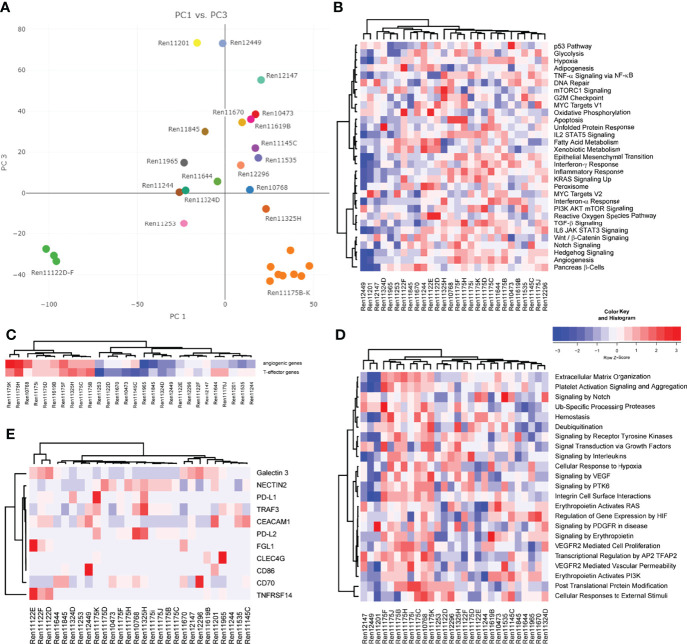
Analysis of gene expression by RNA sequencing of PDX tumor tissue. **(A)** Representation of global gene expression in 28 molecularly characterized RCC PDX models by principal component analysis. The spatial distribution of samples reflects similarity of transcriptomes and correlates with clinical categorization of patient tumor tissue. Colors indicate individual donor patients. Similarity of RCC PDX models in gene set enrichment regarding cancer hallmarks **(B)**, the 66-gene signature distinguishing angiogenic or T-effector type **(C)**, and reactome pathways associated with *VEGF* and *EPO* function **(D)** are visualized by heat map and hierarchical clustering. **(E)** Relative expression of immune checkpoint ligands in the individual PDX tumors, visualized by heat map and hierarchical clustering.

We next aimed to analyze gene expression profiles that could help to distinguish specific RCC subtypes and allow for uncovering of molecular features in individual RCC PDX models corresponding to properties of the human tumor. Principal component analysis (PCA) utilizing overall gene expression of RCC PDX tumors separates the pre-determined disease subtypes indicating considerable expression differences between clear cell (middle), urothelial (top: Ren11201, Ren12449, and Ren12147) and papillary RCC (bottom right: Ren11175B-K) (**Figure 3A**). As expected, multi-tumor PDX models from one and the same patient show very similar overall gene expression as visualized by close proximity in the PCA (Ren11175B-K and Ren11122D-F). Although diagnosed as ccRCC, the transcriptome of Ren11122D-F (bottom left) differs significantly from the other ccRCC PDX models and a deeper analysis could clarify the molecular background of these models. In summary, the PCA plot of this RCC PDX subset reflects the observed clinical heterogeneity in ccRCC disease.

Gene expression data of RCC PDX tumors were subjected to ssGSEA to determine similarities or differences in the enrichment of cancer- as well as pathway-specific gene sets. Scoring and hierarchical clustering of cancer hallmarks clearly differentiated the urothelial RCC models Ren11201, Ren12449, and Ren12147 from other RCC subtypes (**Figure 3B**). These three models were characterized by lower enrichment scores, especially in immune-signaling response pathways, epithelial–mesenchymal transition, KRAS and hedgehog signaling, as well as hypoxia and angiogenesis (**Figure 3B**). The regulation not only of angiogenesis but also of the local immune system greatly impacts RCC development and response to treatment, and the corresponding expression signatures can be used to identify high-risk patients (34). According to the recently published 66-gene signature, the determination of the angiogenic or T-effector type of RCC tumors identifies distinct groups in our PDX cohort (**Figure 3C**). The papillary RCC models show high scores for the angiogenic and the T-effector type, whereas the other models were moderately enriched for only one type. We focused further on the molecular pathways associated with VEGF and erythropoietin signaling and analyzed the models according to their scoring of selected reactome pathways. Again, we observe differential enrichment patterns, clearly separating urothelial RCC and papillary PDX models (mainly low and high enrichment scores, respectively) from the ccRCC models (**Figure 3D**).

Most recent achievements and success with immune checkpoint inhibition in the treatment of RCC (11, 12) result in new challenging requirements for preclinical oncology. Development of humanized RCC PDX models will be a prerequisite for testing of ICIs in mouse models. Gene expression of novel targets for tumor-immune cell crosstalk was analyzed based on a recent review of novel candidates for checkpoint blockade (42). In particular, RCC PDX models demonstrating high transcript expression of immune checkpoint ligands, e.g., as seen for Ren11175K and *PD-L1* or Ren11122E for *FLG1* and *TNFRSF14*, might be considered for future establishment of humanized RCC PDX models and response evaluation to current and/or future ICIs (**Figure 3E**).

Finally, we aimed to further characterize the molecular landscape of our RCC PDX panel investigating potential CNV. Prediction of CNVs from RNAseq data indicated copy number gains and losses for chromosome arms per PDX model (**Supplemental Table S2**). In our panel, the highest number of CNV losses was determined for chromosome 9, with 8 models showing a loss of both chromosome arms, followed by chromosome 13q. High number gains were determined for chromosomes 11, 16, and 19. Loss of chromosomes 3p, 14q, but not 22q, and gains for chromosomes 5q, 7, 16, and 17 were previously published as specific CNV pattern for RCC subtypes (43). Although they derive from the same respective patient, we noticed higher heterogeneity of CNV regions in the papillary RCC models (Ren11175B-K) compared to multi-donor PDX models Ren11122D-E. This was not expected by overall gene expression or gene set enrichment analysis (**Figure 3**).

### Drug Sensitivity Studies

Since various targeted agents for inhibition of the VEGF axis and the PI3K-mTOR signaling pathway were approved for the systemic treatment of RCC, we tested the response of the anti-angiogenic drugs bevacizumab, sunitinib, and sorafenib and the mTOR inhibitor everolimus in our established RCC PDX cohort. Mice were treated for three to five cycles depending on their actual tumor growth in the respective control group. In general, therapeutic treatment of the RCC PDX models revealed a good safety profile and was well tolerated. Minor and reversible BW reduction lower than 10% was noticed in individual animals, but not related to compound-specific toxicity.

The response to each SoC treatment was heterogeneous throughout our RCC PDX panel, reflecting the heterogenic clinical response observed in patients. In brief, sunitinib was the most effective drug in reducing tumor growth, followed by bevacizumab and everolimus. Interestingly, the majority of RCC PDX models from our panel were resistant towards treatment with sorafenib.

**Figure 4:**
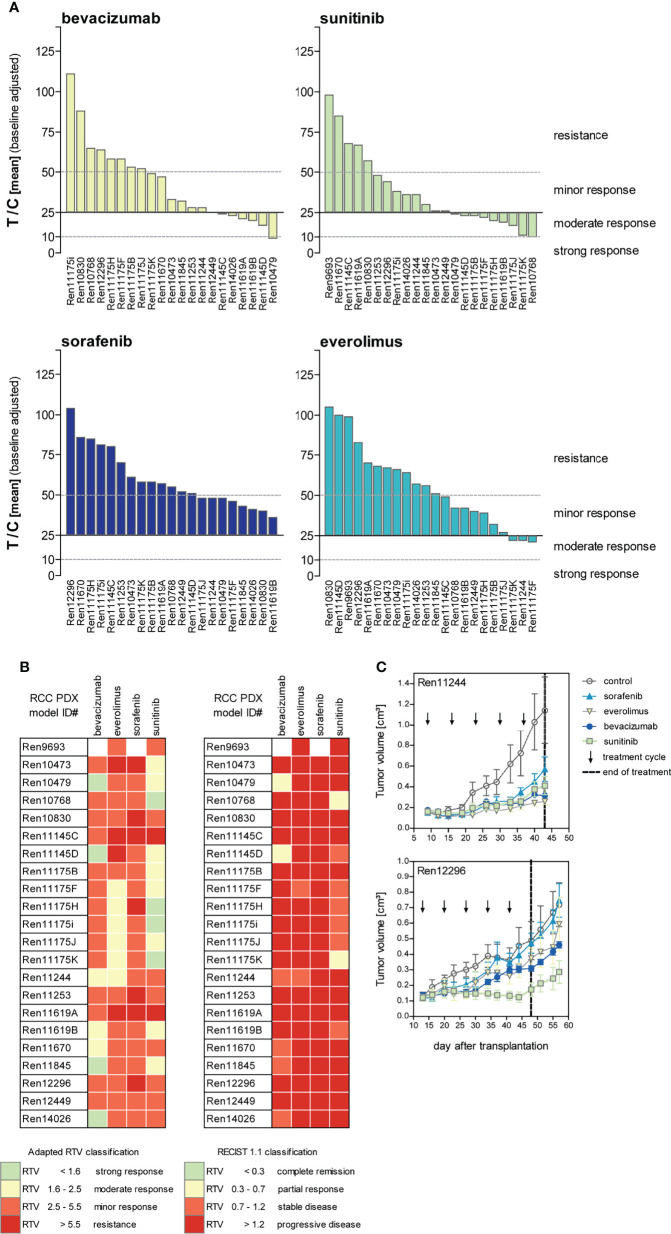
Response characterization of renal cancer PDX models upon treatment with targeted therapies blocking angiogenic and proliferative pathways. **(A)** Mean tumor volume of treated mice compared to the mean tumor volume of control (T/C mean) from 22 PDX sensitivity studies (n = 3–5 individual animals per group) were illustrated in waterfall plots. Bevacizumab and sorafenib were not tested in Ren9693. Response was categorized as T/C: >50% = resistance/progressive disease (PD), <50% >30% = minor response/stable disease (SD), <30% >10% = moderate response/partial response (PR), and <10% = strong response/complete remission (CR) with zero line equal to T/C = 25%. In the 22 analyzed PDX models, treatment response was best for sunitinib, followed by bevacizumab and everolimus. Lowest response rate was observed for sorafenib. **(B)** Individual PDX models exhibit distinct response pattern against selected compounds. Adapted response criteria utilizing RTV (left) opposed to clinical RECIST response classification (right). Adapted RTV response classification mirrors T/C drug response well, whereas clinical RECIST classification yields dramatic lower responses. **(C)** Different response patterns were observed for individual RCC PDX models during drug testing, illustrating RCC heterogeneity and differences of intra-tumoral regions.

The ratio of mean TV of treated animals to mean TV of control animals (T/C mean) is illustrated for the four drugs in twenty-two PDX models of the RCC cohort as a waterfall plot for each experiment at the final study day (**Figure 4A**). As the use of clinical RECIST criteria for solid tumors is not optimal for the characterization of preclinical tumor response in mice, we implemented our previously published adapted response criteria, delineating strong (T/C <10%), moderate (T/C <25%), and minor response (T/C <50%) from resistance (T/C >50%) (44). For better visualization of responding PDX models, we shifted the baseline to 25%. In the tested cohort of 22 RCC PDX models, sunitinib achieved the best overall response rate of 77% of the models with minor to moderate response. A lower response rate was observed for bevacizumab (64%) and everolimus (45%). The least effective response in our panel was seen for sorafenib with only 36%. Applying the adapted RTV response criteria in the test cohort of 22 PDX models (**Figure 4B**, left), sunitinib and bevacizumab achieved a minor to strong response in 91% and 95% of the cases. The RTV-based efficacy of everolimus and sorafenib was 82% and 68%, respectively.

Although RTV evaluation determines increased response for all compounds compared to T/C analysis, the correlation of both results indicates high validity and reproducibility of drug testing in this platform. In contrast, using clinical RECIST criteria for solid tumors would dramatically confound our present findings, resulting in dramatically lower overall response rates in the RCC PDX cohort. Using a RECIST categorization for the drug response *in vivo*, sunitinib achieved stable disease or partial response in 36% of the cases, and a similar efficacy was noticed for bevacizumab with 32%, considering these PDX models as a responder in our setting. In contrast, almost no response was determined for everolimus and sorafenib (only 9% and 0% stable disease, respectively) (**Figure 4B**, right).

The identified individual anti-tumoral response against VEGF RTKI treatments in our RCC PDX panel mirrors the heterogeneity of individual patient tumor characteristics well. Furthermore, our experiments enable the identification of preclinical drug resistance and allow us to distinguish between responsive (“responder”) from resistant (“non-responder”) RCC PDX models. On the one hand, Ren12296 is resistant towards sorafenib, everolimus, and bevacizumab, whereas disease stabilization was observed for sunitinib (**Figure 4C**, lower graph). On the other hand, early disease stabilization was found in Ren11244 for all compounds. However, here, the initial response might convert into disease progression or even development of resistance after several weeks of treatment for single compounds (**Figure 4C**, upper graph). As illustrated for the representative drug response testing graphs (**Figure 4C**), data for all tested RCC PDX models are available in the **Supplemental Material** (**Supplementary Figure S1**).

## Discussion

Clinical approval of targeted therapies resulted in considerable progress for RCC patients requiring systemic treatment. Most recent achievements, by targeting the immune system with ICIs in particular, further extend the portfolio of therapeutic options and significantly improve the progression-free survival of RCC patients. Nevertheless, obstacles remain, considering limited treatment response through molecularly undetermined resistance mechanisms, lack of reliable predictive biomarkers, and frequent clinical complications, leading to treatment-induced drug resistance of local tumors. Even worse, treatment of advanced RCC or refractory tumors with advanced targeted therapies can lead to metastatic progression, representing a major clinical challenge.

For this reason, preclinical research is mandatory to evaluate alternative therapeutic options for subsequent clinical translation. PDX models are considered the gold standard in preclinical oncology, and the use of humanized mouse models would even enable evaluation of immunotherapies. With the current study, we successfully established a molecularly characterized RCC PDX panel as a drug testing platform for this disease. In contrast to previously published RCC PDX cohorts (40), our panel consists of a larger number of models, predominantly from untreated primary tumors and metastases of distinct localizations, and enables the evaluation of ITH on treatment response by multi-regional RCC PDX models. To the best of our knowledge, this was only reported once very recently for RCC PDX models (45) and is a special feature of our RCC PDX panel. As ITH was recently reported to help predict the patient response against PD-1 blockade, and metabolic differences in distinct tumor areas may account for heterogeneity in drug sensitivity, this aspect will gain importance in future anti-cancer therapy (46, 47).

Our newly established PDX panel consisted of forty-six RCC models predominantly derived from untreated primary RCC tumor tissue. The overall take rate of RCC tumors in our cohort was 21%, presumably affected by the treatment status of the donor patient, such as adjuvant or neo-adjuvant therapy. Unfortunately, clinical information on treatment of donor patients was rare. However, no PDX model could be established from the twenty-one patients with disclosed neoadjuvant treatment history (10% sunitinib, 90% everolimus). Nevertheless, our methodology with s.c. tumor fragment transplantation was superior in terms of take rate compared to orthotopic RCC patient-derived tumor graft models (45). A very similar effect of reduced take rates was reported during the establishment of PDX models from breast cancer patients after neoadjuvant therapy (48, 49). In contrast, a higher take rate was achieved for more aggressive triple-negative breast cancer, which goes in line with the observed take rate >80% in more aggressive grade 3 and 4 RCC models in our cohort.

The mean tumor doubling time (TDT) was determined to be larger than 15 days for almost all RCC PDX models. Compared to RCC cell line-derived xenograft (CDX) models, PDX tumors grow significantly slower. Although not specifically reported for ccRCC, a possible bias induced by elevated genetic drift in highly proliferative cell line xenografts compared to PDX models is relatively low and an advantage in the PDX setting (50). Not surprisingly, we observed a wide range of individual growth characteristics similar to what is being observed in patients. In consequence, an appropriate preclinical RCC study design requires greater cohort sizes in PDX models with variable tumor growth rates in order to address this heterogeneity.

For cases with available panel sequencing of primary tumor tissue, the concordance with sequence variations in PDX tumors identified by RNAseq ranged between 75% (*ARID1A*) and 100% (*VHL*). Hence, we demonstrate validity of our PDX panel and established a valuable tool reflecting the clinical RCC mutational landscape. However, the technological differences between panel DNA sequencing and RNA sequencing of the human tumor and the PDX tumor tissue, respectively, might prohibit detection of a greater number of matching mutations in the corresponding tissue samples. Different sequencing depths, variations detected in gene regions not covered by the exome panel, and the fact that not all genes with mutations were expressed may contribute to this low concordance. Certainly, specific sequence variations may be detected in PDX tumors due to subclone selection or occur during the course of PDX passages and might not be present in the initial tumor tissue.

Molecular profiling of the RCC PDX cohort comparably verifies high similarity of multi-donor regions and distinguishes between predetermined RCC subgroups, such as urothelial or papillary vs. clear cell renal cancer. As anticipated, no or only weak correlation between the different RCC PDX models were detected utilizing a hierarchical clustering approach for cancer hallmarks or pathway-specific ssGSEA. Nevertheless, descriptive heatmaps for molecular pathways, including angiogenesis and PI3K-AKT-mTOR signaling, known to be involved in RCC, were mandatory for designing specific biomarker or drug testing studies with novel drug candidates. We exploited most recent approaches for deep molecular characterization in RCC for our cohort and identified features for angiogenic, T-effector, and checkpoint target expression (34, 42), any or all of which might be helpful for future preclinical applications of our PDX models. In particular, biomarker expression of novel immune checkpoint targets will show great promise for PDX model selection in terms of development of humanized mouse models for RCC, but will require validation in further primary RCC tumors. This specific option, so far not available with our actual RCC cohort, would add valuable progress for preclinical testing of ICIs for metastatic and refractory RCC.

Recent results point towards a decisive role of CNVs for development, pathogenesis, and progression of RCC (51, 52). We used our RNAseq-based gene expression and mutation data to predict CNVs in the generated PDX models. Unfortunately, no reliable public RCC reference data for CNV analysis was available. A predicted copy number loss on chromosome 9 observed for distinct PDX models (**Supplemental Table S2**, blue) might be related to alterations of *CDKN2A* or *ADOLB* gene expression, which were known to be of clinical relevance in metastatic ccRCC (53). The correlation of predicted CNVs with mutational data and/or functional outcome of the detected region might enable the identification of relevant RCC biomarkers.

Our second main aim in this study was to collect valuable drug testing data for targeted therapies with strong clinical implication for RCC. The optimal classification strategy of treatment response in preclinical oncology is often under debate, but an appropriate study design allows for direct comparison between individual studies independent of the used read-out parameter (54). We choose to report tumor response by the use of recently published adapted response criteria (T/C and RTV) on the final study day for each PDX drug test experiment. Not surprisingly, the *in vivo* tumor response observed with the tested targeted therapies was similar for both read-out parameters.

Sunitinib was superior to other VEGF axis targeting drugs bevacizumab and sorafenib and also to monotherapy with everolimus. Admittedly, in some cases, the applied concentration of selected drugs differs from clinically used dosage. This criterion is a frequent limitation in preclinical studies due to biological differences between the species including plasma binding affinity, IC_50_, and drug metabolism. However, the dosage used in our study was thoroughly selected, balancing efficacy and tolerability *in vivo* and particularly in the used mouse strains. Furthermore, direct comparison with other preclinical studies has guidance character only, since the drug response mainly relies on the molecular phenotype of the tested PDX model (55, 56). However, ideally, the PDX model drug response should match the corresponding response in donor patients. Unfortunately, several of our PDX models were established from primary and untreated tumor biopsies, and data on follow-up were limited. It is worth mentioning that, after biopsy retrieval, a female patient (Ren12723) was treated with sunitinib and responded well towards this treatment, which was also observed *in vivo* for the corresponding PDX model. The resistant PDX Ren11619A did not respond to any of the tested drugs, and a similar renal cancer progressed without any appreciable therapy effect of pazopanib and sunitinib on the donor patient. Moreover, and in line with data from our PDX models, patients Ren11145 and Ren11324 showed clinical disease progression after treatment with sunitinib and sorafenib, followed by everolimus and sunitinib, respectively. Therefore, the correlation of VEGF RTKI response in RCC PDX models and patient-specific clinical response can be reported for these four cases providing evidence for general translation of prognostic PDX response data (57).

Finally, the combination of deep molecular sequencing PDX information with a classification of responder vs. non-responder would enable further analysis for the identification of a compound-specific mode of action. Therefore, our dataset possesses a unique predictive power for the translation of preclinical results into clinical options to overcome resistance in RCC.

In order to further expand our preclinical pipeline, we aim to use our RCC PDX panel for the development of a 3D-organoid patient-derived platform facilitating *ex vivo* drug testing assays. Compared to already existing single *in vitro* approaches (58, 59), our combined tool and biostatistics will enable us to increase the predictive power of our patient-derived dataset for the improvement of personalized cancer treatments. In particular, more extensive combination of PDX and *in vitro* testing of non-responsive xenograft tumors might yield novel therapeutic opportunities for RCC patient tumors with comparable molecular characteristics, at least for specific compound classes. Unfortunately, this will most likely not be feasible for the current class of VEGFR inhibitors since their anti-tumoral effect is mainly driven through the antiangiogenic properties of these compounds, as seen for regorafenib, and effective *in vitro* models addressing the underlying mode of action are still lacking (60).

In theory, the use of personalized PDX models will guide oncologists in selecting the best possible treatment option for the corresponding donor patient. In practice, the time span needed to generate and treat the respective model hinders a fast return of individual response prediction for the patient. Nevertheless, large panels of PDX models will reflect the heterogeneity of molecular and pathologic features of the disease and are of great value for the identification and experimental validation of predictive biomarkers and of novel treatment options, such as drug combinations and disease-specific target therapies.

## Data Availability Statement

All underlying RNA sequencing and genome-wide transcriptome data has been deposited at the European Genome-phenome Archive (EGA) (https://ega-archive.org), which is hosted by the EBI and the CRG, under accession numbers EGAS00001006249 and EGAS00001001176, respectively. Further information about EGA can be found on https://ega-archive.org.

## Ethics Statement

The studies involving human participants were reviewed and approved by the local ethics committees of the Otto-von-Guericke University Magdeburg (87/11) and the University of Tübingen (622/2020BO), Germany. The patients/ participants provided their written informed consent to participate in this study before tissue collection and execution of experiments. The animal studies were reviewed and approved by local authorities (Landesamt für Gesundheit und Soziales, LaGeSo Berlin, Germany) under approval number H0032-09 for in vivo PDX passages and A0452-08 for preclinical sensitivity experiments.

## Author Contributions

DG, SF, BJ, WW, and JH designed this work. CS, JB, MatS, JW, MarS, and BJ were responsible for patient tumor tissue collection and inclusion for the project. SF supervised the experimental studies. MB, FB, ES, RB, BJ, MD, and DG analyzed data. DG, WW, and JH wrote the manuscript. All authors contributed to the article and approved the submitted version.

## Funding

This work was supported by the European Union under the seventh framework programme: Predicting individual response and resistance to cancer therapy. FP7-HEALTH-2010-two-stage (Grant agreement ID: 259303). The work was supported by the Robert Bosch Stiftung (Stuttgart, Germany) and the Deutsche Forschungsgemeinschaft (DFG, German Research Foundation) under Germany’s Excellence Strategy—EXC 2180—390900677). JB declares that he has received institutional research funding from AstraZeneca, Astellas, BMS, Eisai, Ipsen, MSD, Novartis, Nektar, Pfizer, Roche, and Seattle Genetics; received honoraria from BMS and MSD on an institutional basis and from AstraZeneca, Astellas, BMS, Eisai, EUSA Pharma, Ipsen, MSD, Merck Serono, Novartis, Pfizer, and Roche on a personal basis. The funders were not involved in the study design, collection, analysis, interpretation of data, the writing of this article or the decision to submit it for publication.

## Conflict of Interest

DG, MB, MD and SF were employed by the company EPO. WW was CSO and JH was CEO of the company EPO.

The remaining authors declare that the research was conducted in the absence of any commercial or financial relationships that could be construed as a potential conflict of interest

## Publisher’s Note

All claims expressed in this article are solely those of the authors and do not necessarily represent those of their affiliated organizations, or those of the publisher, the editors and the reviewers. Any product that may be evaluated in this article, or claim that may be made by its manufacturer, is not guaranteed or endorsed by the publisher.

## References

[1] Shuch B Vourganti S Ricketts CJ Middleton L Peterson J Merino MJ. Defining Early-Onset Kidney Cancer: Implications for Germline and Somatic Mutation Testing and Clinical Management. J Clin Oncol (2014) 32:431–7. doi: 10.1200/JCO.2013.50.8192 PMC3912328 24378414

[2] Moch H Cubilla AL Humphrey PA Reuter VE Ulbright TM. The 2016 WHO Classification of Tumours of the Urinary System and Male Genital Organs—Part A: Renal, Penile, and Testicular Tumours. Eur Urol (2016) 70(1):93–105. doi: 10.1016/j.eururo.2016.02.029 26935559

[3] Linehan WM Ricketts CJ. The Cancer Genome Atlas of Renal Cell Carcinoma: Findings and Clinical Implications. Nat Rev Urol(2019) 16:539–52. doi: 10.1038/s41585-019-0211-5 31278395

[4] Lindgren D Eriksson P Krawczyk K Nilsson H Hansson J Veerla S. Cell-Type-Specific Gene Programs of the Normal Human Nephron Define Kidney Cancer Subtypes. Cell Rep(2017) 20(6):1476–89. doi: 10.1016/j.celrep.2017.07.043 28793269

[5] Latif F Tory K Gnarra J Yao M Duh FM Orcutt ML. Identification of the Von Hippel-Lindau Disease Tumor Suppressor Gene. Science (1993) 260:1317–20. doi: 10.1126/science.8493574 8493574

[6] Kim H Shim BY Lee SJ Lee JY Lee HJ Kim IH. Loss of Von Hippel-Lindau (VHL) Tumor Suppressor Gene Function: VHL-HIF Pathway and Advances in Treatments for Metastatic Renal Cell Carcinoma (RCC). Int J Mol Sci (2021) 22:9795. doi: 10.3390/IJMS22189795 34575959 PMC8470481

[7] Motzer RJ Hutson TE Tomczak P Michaelson MD Bukowski RM Rixe O. Sunitinib Versus Interferon Alfa in Metastatic Renal-Cell Carcinoma. N Engl J Med (2007) 356:115–24. doi: 10.1056/NEJMOA065044 17215529

[8] Kane RC Farrell AT Saber H Tang S Williams G Jee JM. Sorafenib for the Treatment of Advanced Renal Cell Carcinoma. Clin Cancer Res (2006) 12:7271–8. doi: 10.1158/1078-0432.CCR-06-1249 17189398

[9] Motzer RJ Escudier B Oudard S Hutson TE Porta C Bracarda S. Efficacy of Everolimus in Advanced Renal Cell Carcinoma: A Double-Blind, Randomised, Placebo-Controlled Phase III Trial. Lancet (London England) (2008) 372:449–56. doi: 10.1016/S0140-6736(08)61039-9 18653228

[10] Hudes G Carducci M Tomczak P Dutcher J Figlin R Kapoor A. Temsirolimus, Interferon Alfa, or Both for Advanced Renal-Cell Carcinoma. (2009) 356:2271–81. doi: 10.1056/NEJMOA066838 17538086

[11] Powles T Plimack ER Soulières D Waddell T Stus V Gafanov R. Pembrolizumab Plus Axitinib Versus Sunitinib Monotherapy as First-Line Treatment of Advanced Renal Cell Carcinoma (KEYNOTE-426): Extended Follow-Up From a Randomised, Open-Label, Phase 3 Trial. Lancet Oncol(2020) 21:1563–73. doi: 10.1016/S1470-2045(20)30436-8 33284113

[12] Choueiri TK Powles T Burotto M Escudier B Bourlon MT Zurawski B. Nivolumab Plus Cabozantinib Versus Sunitinib for Advanced Renal-Cell Carcinoma. N Engl J Med(2021) 384:829–41. doi: 10.1056/nejmoa2026982/suppl_file/nejmoa2026982_data-sharing.pdf PMC8436591 33657295

[13] Bedke J Albiges L Capitanio U Giles RH Hora M Lam TB. The 2021 Updated European Association of Urology Guidelines on Renal Cell Carcinoma: Immune Checkpoint Inhibitor-Based Combination Therapies for Treatment-Naive Metastatic Clear-Cell Renal Cell Carcinoma Are Standard of Care. Eur Urol (2021) 80:393–7. doi: 10.1016/J.EURURO.2021.04.042 34074559

[14] Jamal-Hanjani M Quezada SA Larkin J Swanton C. Translational Implications of Tumor Heterogeneity. Clin Cancer Res(2015) 21:1258–66. doi: 10.1158/1078-0432.CCR-14-1429 PMC4374162 25770293

[15] Rini BI Atkins MB. Resistance to Targeted Therapy in Renal-Cell Carcinoma. Lancet Oncol(2009) 10:992–1000. doi: 10.1016/S1470-2045(09)70240-2 19796751

[16] Choueiri TK Escudier B Powles T Mainwaring PN Rini BI Donskov F. Cabozantinib Versus Everolimus in Advanced Renal-Cell Carcinoma. N Engl J Med (2015) 373:1814–23. doi: 10.1056/nejmoa1510016/suppl_file/nejmoa1510016_disclosures.pdf PMC5024539 26406150

[17] Motzer R Alekseev B Rha S-Y Porta C Eto M Powles T. Lenvatinib Plus Pembrolizumab or Everolimus for Advanced Renal Cell Carcinoma. N Engl J Med (2021) 384:1289–300. doi: 10.1056/nejmoa2035716/suppl_file/nejmoa2035716_data-sharing.pdf 33616314

[18] Heng DYC Xie W Regan MM Warren MA Golshayan AR Sahi C. Prognostic Factors for Overall Survival in Patients With Metastatic Renal Cell Carcinoma Treated With Vascular Endothelial Growth Factor-Targeted Agents: Results From a Large, Multicenter Study. J Clin Oncol (2009) 27:5794–9. doi: 10.1200/JCO.2008.21.4809 19826129

[19] Fichtner I Klinghammer K Behrens D Flechsig S Rolff J Becker M. Animal Models for Personalized Treatment Options. Int J Clin Pharmacol Ther (2017) 55:698–700. doi: 10.5414/cpxces15ea09 28696202

[20] Langdon SP Hendriks HR Braakhuis BJ Pratesi G Berger DP Fodstad O. Preclinical Phase II Studies in Human Tumor Xenografts: A European Multicenter Follow-Up Study. Ann Oncol(1994) 5:415–22. doi: 10.1093/oxfordjournals.annonc.a058872 8075048

[21] Klinghammer K Raguse JD Plath T Albers AE Joehrens K Zakarneh A. A Comprehensively Characterized Large Panel of Head and Neck Cancer Patient-Derived Xenografts Identifies the mTOR Inhibitor Everolimus as Potential New Treatment Option. Int J Cancer (2015) 136:2940–8. doi: 10.1002/IJC.29344 25404014

[22] Fichtner I Rolff J Soong R Hoffmann J Hammer S Sommer A. Establishment of Patient-Derived Non-Small Cell Lung Cancer Xenografts as Models for the Identification of Predictive Biomarkers. Clin Cancer Res (2008) 14:6456–68. doi: 10.1158/1078-0432.CCR-08-0138 18927285

[23] Rolff J Becker M Merk J Hoffmann J Fichtner I. Preclinical Study of a Combination of Erlotinib and Bevacizumab in Early Stages of Unselected Non-Small Cell Lung Cancer Patient-Derived Xenografts. Tar Oncol (2016) 11:507–14. doi: 10.1007/s11523-015-0415-4 26817645

[24] Workman P Aboagye EO Balkwill F Balmain A Bruder G Chaplin DJ. Guidelines for the Welfare and Use of Animals in Cancer Research. Br J Cancer (2010) 102:1555–77. doi: 10.1038/sj.bjc.6605642 PMC2883160 20502460

[25] Nair A Jacob S. A Simple Practice Guide for Dose Conversion Between Animals and Human. J Basic Clin Pharm (2016) 7(2):27–31. doi: 10.4103/0976-0105.177703 27057123 PMC4804402

[26] Andrew S. FastQC: A Quality Control Tool for High Throughput Sequence Data (2018). Available at: https://www.bioinformatics.babraham.ac.uk/projects/fastqc.

[27] Conway T Wazny J Bromage A Tymms M Sooraj D Williams ED. Xenome–A Tool for Classifying Reads From Xenograft Samples. Bioinformatics(2012) 28:i172–8. doi: 10.1093/bioinformatics/bts236 PMC3371868 22689758

[28] Dobin A Davis CA Schlesinger F Drenkow J Zaleski C Jha S. STAR: Ultrafast Universal RNA-Seq Aligner. Bioinformatics (2013) 29:15–21. doi: 10.1093/bioinformatics/bts635 23104886 PMC3530905

[29] Okonechnikov K Conesa A Garcia-Alcalde F. Qualimap 2: Advanced Multi-Sample Quality Control for High-Throughput Sequencing Data. Bioinformatics(2016) 32:292–4. doi: 10.1093/bioinformatics/btv566 PMC4708105 26428292

[30] Roberts Pachter L. Express. A. Streaming Quantification for High-Throughput Sequencing. Available at: https://pachterlab.github.io/eXpress/overview.html.

[31] Smid M Coebergh van den Braak RRJ van de Werken HJG van Riet J van Galen A de Weerd V. Gene Length Corrected Trimmed Mean of M-Values (GeTMM) Processing of RNA-Seq Data Performs Similarly in Intersample Analyses While Improving Intrasample Comparisons. BMC Bioinf(2018) 19:236. doi: 10.1186/S12859-018-2246-7 PMC6013957 29929481

[32] Krug K Mertins P Zhang B Hornbeck P Raju R Ahmad R. A Curated Resource for Phosphosite-Specific Signature Analysis. Mol Cell Proteomics (2019) 18:576–93. doi: 10.1074/MCP.TIR118.000943 PMC6398202 30563849

[33] Liberzon A Birger C Thorvaldsdóttir H Ghandi M Mesirov JP Tamayo P. The Molecular Signatures Database (MSigDB) Hallmark Gene Set Collection. Cell Syst (2015) 1:417–25. doi: 10.1016/J.CELS.2015.12.004 PMC4707969 26771021

[34] D’Costa NM Cina D Shrestha R Bell RH Lin YY Asghari H. Identification of Gene Signature for Treatment Response to Guide Precision Oncology in Clear-Cell Renal Cell Carcinoma. Sci Rep (2020) 10:2026. doi: 10.1038/S41598-020-58804-Y 32029828 PMC7005147

[35] Genome Analysis Toolkit Variant Discovery in High-Throughput Sequencing Data. Available at: https://gatk.broadinstitute.org/hc/en-us.

[36] McLaren W Gil L Hunt SE Riat HS Ritchie GRS Thormann A. The Ensembl Variant Effect Predictor. Genome Biol(2016) 17:1–14. doi: 10.1186/S13059-016-0974-4/TABLES/8 27268795 PMC4893825

[37] Sauter-Meyerhoff C Bohnert R Mazzola P Stühler V Kandabarau S Büttner FA. Characterization of Genetic Heterogeneity in Recurrent Metastases of Renal Cell Carcinoma. Cancers (Basel)(2021) 13:6221. doi: 10.3390/CANCERS13246221 34944839 PMC8699544

[38] Winter S Fisel P Büttner F Rausch S D’Amico D Hennenlotter J. Methylomes of Renal Cell Lines and Tumors or Metastases Differ Significantly With Impact on Pharmacogenes. Sci Rep (2016) 6:29930. doi: 10.1038/SREP29930 27435027 PMC4951699

[39] Büttner F Winter S Rausch S Reustle A Kruck S Junker K. Survival Prediction of Clear Cell Renal Cell Carcinoma Based on Gene Expression Similarity to the Proximal Tubule of the Nephron. Eur Urol (2015) 68:1016–20. doi: 10.1016/J.EURURO.2015.05.045 26072688

[40] Tracey AT Murray KS Coleman JA Kim K. Patient-Derived Xenograft Models in Urological Malignancies: Urothelial Cell Carcinoma and Renal Cell Carcinoma. Cancers (Basel) (2020) 12:439. doi: 10.3390/CANCERS12020439 PMC7072311 32069881

[41] Creighton CJ Morgan M Gunaratne PH Wheeler DA Gibbs RA Robertson G. Comprehensive Molecular Characterization of Clear Cell Renal Cell Carcinoma. Nat(2013) 499:43–9. doi: 10.1038/nature12222 PMC3771322 23792563

[42] Qin S Xu L Yi M Yu S Wu K Luo S. Novel Immune Checkpoint Targets: Moving Beyond PD-1 and CTLA-4. Mol Cancer (2019) 18:155. doi: 10.1186/S12943-019-1091-2 31690319 PMC6833286

[43] Ricketts CJ De Cubas AA Fan H Smith CC Lang M Reznik E. The Cancer Genome Atlas Comprehensive Molecular Characterization of Renal Cell Carcinoma. Cell Rep(2018) 23:313–26. doi: 10.1016/j.celrep.2018.03.075 PMC6075733 29617669

[44] Keil M Conrad T Becker M Keilholz U Yaspo ML Lehrach H. Modeling of Personalized Treatments in Colon Cancer Based on Preclinical Genomic and Drug Sensitivity Data. Cancers (Basel) (2021) 13, 6018. doi: 10.3390/CANCERS13236018 34885128 PMC8656546

[45] Elias R Tcheuyap VT Kaushik AK Singla N Gao M Reig Torras O. A Renal Cell Carcinoma Tumorgraft Platform to Advance Precision Medicine. Cell Rep(2021) 37(8):110055. doi: 10.1016/j.celrep.2021.110055 34818533 PMC8762721

[46] Ran X Xiao J Zhang Y Teng H Cheng F Chen H. Low Intratumor Heterogeneity Correlates With Increased Response to PD-1 Blockade in Renal Cell Carcinoma. Ther Adv Med Oncol(2020) 12:1758835920977117. doi: 10.1177/1758835920977117 33425025 PMC7758866

[47] Okegawa T Morimoto M Nishizawa S Kitazawa S Honda K Araki H. Intratumor Heterogeneity in Primary Kidney Cancer Revealed by Metabolic Profiling of Multiple Spatially Separated Samples Within Tumors. EBioMedicine (2017) 19:31–8. doi: 10.1016/J.EBIOM.2017.04.009 PMC5440602 28408240

[48] Goetz MP Kalari KR Suman VJ Moyer AM Yu J Visscher DW. Tumor Sequencing and Patient-Derived Xenografts in the Neoadjuvant Treatment of Breast Cancer. JNCI J Natl Cancer Inst(2017) 109(7):djw306. doi: 10.1093/JNCI/DJW306 PMC5408989 28376176

[49] Yu J Qin B Moyer AM Sinnwell JP Thompson KJ Copland JA. Establishing and Characterizing Patient-Derived Xenografts Using Pre-Chemotherapy Percutaneous Biopsy and Post-Chemotherapy Surgical Samples From a Prospective Neoadjuvant Breast Cancer Study. Breast Cancer Res(2017) 19:130. doi: 10.1186/S13058-017-0920-8 29212525 PMC5719923

[50] Wolf MM Kimryn Rathmell W Beckermann KE. Modeling Clear Cell Renal Cell Carcinoma and Therapeutic Implications. Oncogene (2020) 39:3413. doi: 10.1038/S41388-020-1234-3 32123314 PMC7194123

[51] Krill-Burger JM Lyons MA Kelly LA Sciulli CM Petrosko P Chandran UR. Renal Cell Neoplasms Contain Shared Tumor Type-Specific Copy Number Variations. Am J Pathol (2012) 180:2427–39. doi: 10.1016/j.ajpath.2012.01.044 PMC3378847 22483639

[52] Zhou W Yang F Xu Z Luo M Wang P Guo Y. Comprehensive Analysis of Copy Number Variations in Kidney Cancer by Single-Cell Exome Sequencing. Front Genet (2020) 10:1379/BIBTEX. doi: 10.3389/FGENE.2019.01379/BIBTEX 32038722 PMC6989475

[53] Nouhaud FX Blanchard F Sesboue R Flaman JM Sabourin JC Pfister C. Clinical Relevance of Gene Copy Number Variation in Metastatic Clear Cell Renal Cell Carcinoma. Clin Genitourin Cancer (2018) 16:e795–805. doi: 10.1016/J.CLGC.2018.02.013 29548613

[54] Malcolm JE Stearns TM Airhart SD Graber JH Bult CJ. Factors That Influence Response Classifications in Chemotherapy Treated Patient-Derived Xenografts (PDX). PeerJ(2019) 7:e6586. doi: 10.7717/peerj.6586 30944774 PMC6441558

[55] Schueler J Klingner K Bug D Zoeller C Maier A Dong M. Patient Derived Renal Cell Carcinoma Xenografts Exhibit Distinct Sensitivity Patterns in Response to Antiangiogenic Therapy and Constitute a Suitable Tool for Biomarker Development. Oncotarget(2018) 9:30946–61. doi: 10.18632/ONCOTARGET.25697 PMC6089561 30123419

[56] Lang H Béraud C Bethry A Danilin S Lindner V Coquard C. Establishment of a Large Panel of Patient-Derived Preclinical Models of Human Renal Cell Carcinoma. Oncotarget (2016) 7:59336–59. doi: 10.18632/ONCOTARGET.10659 PMC5312316 27449081

[57] Dong Y Manley BJ Becerra MF Redzematovic A Casuscelli J Tennenbaum DM. Tumor Xenografts of Human Clear Cell Renal Cell Carcinoma But Not Corresponding Cell Lines Recapitulate Clinical Response to Sunitinib: Feasibility of Using Biopsy Samples. Eur Urol Focus (2017) 3:590–8. doi: 10.1016/J.EUF.2016.08.005 PMC5608640 28753786

[58] Esser LK Branchi V Leonardelli S Pelusi N Simon AG Klümper N. Cultivation of Clear Cell Renal Cell Carcinoma Patient-Derived Organoids in an Air-Liquid Interface System as a Tool for Studying Individualized Therapy. Front Oncol (2020) 10:1775. doi: 10.3389/fonc.2020.01775 33072556 PMC7537764

[59] Kazama A Anraku T Kuroki H Shirono Y Murata M Bilim V. Development of Patient-Derived Tumor Organoids and a Drug Testing Model for Renal Cell Carcinoma. Oncol Rep(2021) 46:226. doi: 10.3892/or.2021.8177 34468011 PMC8424486

[60] Vlachogiannis G Hedayat S Vatsiou A Jamin Y Fernández-Mateos J Khan K. Patient-Derived Organoids Model Treatment Response of Metastatic Gastrointestinal Cancers. Science (2018) 359:920–6. doi: 10.1126/SCIENCE.AAO2774 PMC6112415 29472484

