# Supplementary material for: A Molecularly Characterized Preclinical Platform of Subcutaneous Renal Cell Carcinoma (RCC) Patient-Derived Xenograft Models to Evaluate Novel Treatment Strategies
Source: Front Oncol. 2022 Jun 21;12:889789. doi: 10.3389/fonc.2022.889789 (PMC9254864; doi:10.3389/fonc.2022.889789)
Supplement: Supplementary file 1 [file DataSheet_1.pdf]

Supplementary Material

Supplementary Figure S1: Response characterization of renal cancer PDX models upon treatment with targeted therapies blocking angiogenic and proliferative pathways

(corresponding to exemplary graphs in Figure 4c)

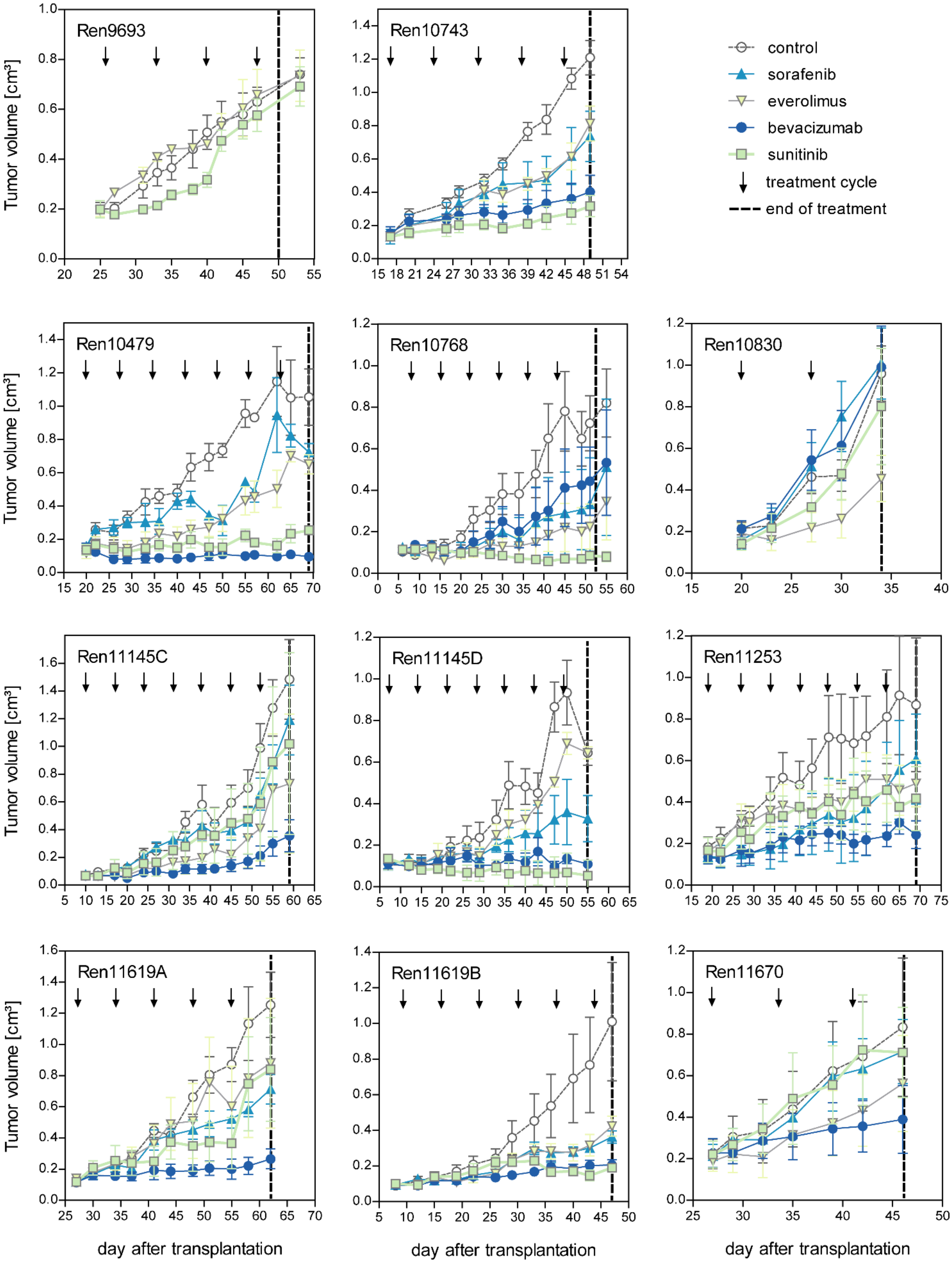

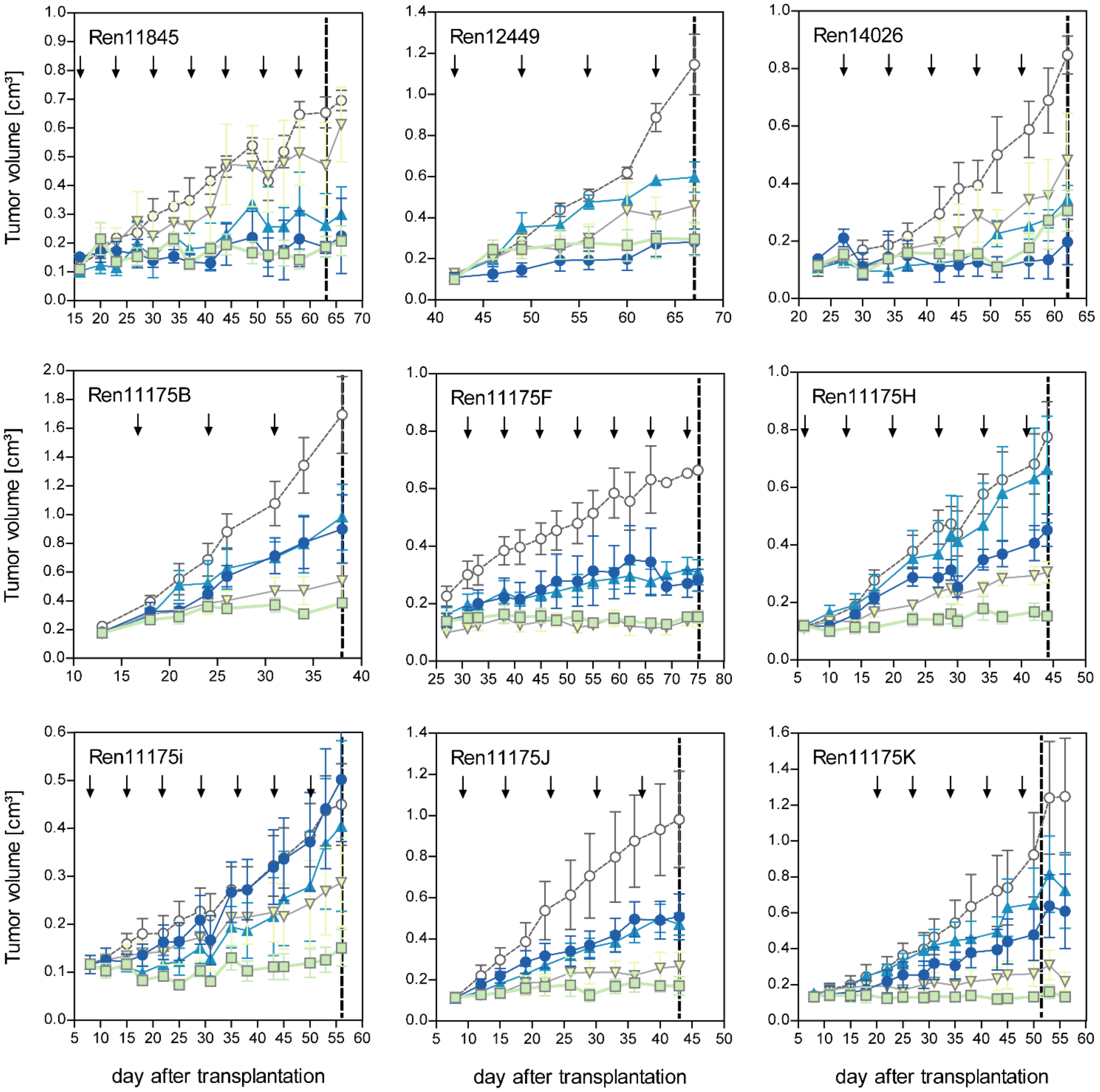

Figure S1: Response characterization of renal cancer PDX models **(corresponding to exemplary graphs in Figure 4c)**

Different response pattern were observed for individual RCC PDX models during drug testing illustrating RCC heterogeneity and differences of intra-tumoral regions. Results from 20 PDX sensitivity studies (n = 3 - 5 individual animals per group). Bevacizumab and sorafenib were not tested in Ren9693. Response was categorized as T/C: > 50% = resistant, < 50% > 30% = minor response, <30% >10% = moderate response and < 10% = strong response with zero line equal to T/C=25%.

Supplementary Table 1 (Table S1): Somatic mutations detected in RCC PDX by RNASeq corresponding to Figure 2a

RCC PDX model ID	Gene name	Chromosome (CHROM)	Position (POS)	Reference base(s) (REF)	Alternate base(s) (ALT)	Amino acid change	Variant class	Functional consequence	dbSNP ID[1]	PolyPhen-2 prediction[2]	SIFT prediction[3]
Ren10473	MTOR	1	11128021	C	A	V2006F	SNV	missense variant	-	probably damaging	deleterious
Ren10473	TP53	17	7673790	C	A	C277F	SNV	missense variant	rs763098116	probably damaging	deleterious
Ren10473	VHL	3	10142041	C	A	S65*	SNV	stop gained	rs5030826	-	-
Ren11122F	TP53	17	7675217	T	C	K132R	SNV	missense variant	rs1057519996	probably damaging	deleterious
Ren11145C	PBRM1	3	52658315	A	ATCCTTCAGTCACTGTGCCCTGATTGTCTTGCCCATCTTCATCATCATCTTCGTCATCTGCTT	-206-207KQMTKMMMKMGKTIRAQ*LKX	insertion	stop gained frameshift variant	-	-	-
Ren11145C	VHL	3	10142057	GC	G	P71X	deletion	frameshift variant	-	-	-
Ren11175B	KDM5C	X	53193842	A	C	L1350V	SNV	missense variant	-	benign	tolerated
Ren11175B	MET	7	116769777	G	T	E925*	SNV	stop gained	-	-	-
Ren11175B	NF2	22	29671847	C	T	R341*	SNV	stop gained	rs74315499	-	-
Ren11175B	PBRM1	3	52658315	A	ATCCTTCAGTCACTGTGCCCTGATTGTCTTGCCCATCTTCATCATCATCTTCGTCATCTGCTTCTCCTTTCTGAACAAACTCATTTCTTGTT	-206-207NKK*VCSERRSR*RR***RWARQSGHSD*RX	insertion	stop gained frameshift variant	-	-	-
Ren11175C	NF2	22	29671847	C	T	R341*	SNV	stop gained	rs74315499	-	-
Ren11175F	NF2	22	29671847	C	T	R341*	SNV	stop gained	rs74315499	-	-
Ren11175F	PBRM1	3	52658315	A	ATC	-206-207X	insertion	frameshift variant	-	-	-

Ren11175H	ARID1A	1	26696755	A	C	T118P	SNV	missense variant	-	benign	tolerated low confidence
Ren11175H	NF2	22	29671847	C	T	R341*	SNV	stop gained	rs74315499	-	-
Ren11175H	RB1	13	48459796	A	G	N690S	SNV	missense variant	-	possibly damaging	deleterious
Ren11175H	TCEB1	8	73956046	C	A	E5*	SNV	stop gained	-	-	-
Ren11175i	NF2	22	29671847	C	T	R341*	SNV	stop gained	rs74315499	-	-
Ren11175J	NF2	22	29671847	C	T	R341*	SNV	stop gained	rs74315499	-	-
Ren11175K	RB1	13	48476796	G	GA	-872-873X	insertion	frameshift variant	-	-	-
Ren11201	ARID1A	1	26774515	C	T	Q1430*	SNV	stop gained	-	-	-
Ren11201	KDM5C	X	53195061	C	G	C1103S	SNV	missense variant	-	benign	tolerated
Ren11201	PBRM1	3	52658315	A	ATC	-206-207X	insertion	frameshift variant	-	-	-
Ren11244	BAP1	3	52403559	T	TAAGGGCAGAGTTGGTGTTCTGCACGTCATCCTCCTCGTCATCCTCATAGTCATCCTCATCATCTGAGTACTGC	K529SSTQMMRMTMRMTRRMTCRTPTLPLX	insertion	frameshift variant	-	-	-
Ren11244	BAP1	3	52405270	G	C	S319*	SNV	stop gained	-	-	-
Ren11244	TSC1	9	132897301	CTT	C	KR952-953KX	deletion	frameshift variant	-	-	-
Ren11253	ARID1A	1	26696422	CCCGCCGCCGCCAGCAGCCTGGGCAACCCGCCG	C	PAAASSLGNPP7-17X	deletion	frameshift variant	-	-	-
Ren11253	MTOR	1	11133097	C	T	D1783N	SNV	missense variant	rs1471906651	probably damaging	tolerated
Ren11324D	ARID1A	1	26779998	G	T	E2034*	SNV	stop gained	-	-	-
Ren11325H	ARID1A	1	26696755	A	C	T118P	SNV	missense variant	-	benign	tolerated low confidence
Ren11325H	KDM5C	X	53210820	G	A	P480L	SNV	missense variant	rs1057518697	probably damaging	deleterious
Ren11325H	SETD2	3	47017244	C	G	G2541A	SNV	missense variant	-	-	-

Ren11325H	TSC1	9	132897563	G	GT	N891KX	insertion	frameshift variant	rs118203724	-	-
Ren11325H	VHL	3	10149811	T	C	L163P	SNV	missense variant	rs28940297	probably damaging	deleterious
Ren11535	ARID1A	1	26696755	A	C	T118P	SNV	missense variant	-	benign	tolerated low confidence
Ren11535	ARID1A	1	26697156	GCCTC	G	PP252-253X	deletion	frameshift variant	-	-	-
Ren11535	VHL	3	10142087	T	G	S80R	SNV	missense variant	-	probably damaging	deleterious
Ren11619B	BAP1	3	52403439	ACCCC	A	GV568-569X	deletion	frameshift variant	-	-	-
Ren11619B	FH	1	241500571	G	A	S419L	SNV	missense variant	rs1131691244	probably damaging	deleterious
Ren11619B	KMT2C	7	152156216	A	G	V4006A	SNV	missense variant	rs775922637	-	-
Ren11619B	MET	7	116699799	C	G	P258A	SNV	missense variant	-	-	-
Ren11619B	MET	7	116782048	C	T	L1214F	SNV	missense variant	rs121913673	-	-
Ren11644	KDM5C	X	53210733	G	A	S509L	SNV	missense variant	-	probably damaging	deleterious
Ren11644	TCEB1	8	73946815	A	T	F52I	SNV	missense variant	-	possibly damaging	deleterious
Ren11670	VHL	3	10142149	T	C	L101P	SNV	missense variant	-	benign	deleterious
Ren11845	STK11	19	1228059	C	CT	L594LX	insertion	frameshift variant	-	-	-
Ren11845	VHL	3	10142149	T	C	L101P	SNV	missense variant	-	benign	deleterious
Ren11965	MTOR	1	11247947	A	T	L330M	SNV	missense variant	-	benign	tolerated
Ren12147	PTEN	10	87957999	C	T	Q434*	SNV	stop gained	rs730882131	-	-
Ren12147	STAG2	X	124071311	A	G	N841D	SNV	missense variant	-	benign	tolerated
Ren12147	TP53	17	7674211	A	T	I251N	SNV	missense variant	-	probably damaging	deleterious
Ren12449	ARID1A	1	26696671	A	G	S90G	SNV	missense variant	rs752026201	benign	tolerated low confidence

Ren12449	KMT2C	7	152235912	C	T	G893R	SNV	missense variant	rs199831680	-	-
Ren12449	KMT2C	7	152247986	G	GT	Y817*	insertion	stop gained frameshift variant	rs150073007	-	-
Ren12449	KMT2C	7	152248119	G	A	S773L	SNV	missense variant	rs4024453	-	-

Table S1: Somatic mutations detected in RCC PDX by RNASeq corresponding to Figure 2a

Using RNA sequencing data, 31 genes that are frequently mutated in RCC were analyzed for somatic alterations. The variants shown were either not included in gnomAD 3.1 or had allele frequencies below 0.0001. Variants were annotated with Ensembl Variant Effect Predictor (VEP) Release 94 (1)

1 https://www.ncbi.nlm.nih.gov/snp/

2 http://genetics.bwh.harvard.edu/pph2/

3 http://sift-dna.org

1. Liberzon A, Birger C, Thorvaldsdóttir H, Ghandi M, Mesirov JP, Tamayo P. The Molecular Signatures Database (MSigDB) hallmark gene set collection. *Cell Syst* (2015) **1**:417–425. doi:10.1016/J.CELS.2015.12.004

Supplementary Table 2 (Table S2): Prediction of CNVs from RNAseq

PDX model / chromosome (arm)	1		2		3		4		5		6		7		8		9		10		11		12		13	14	15	16		17		18		19		20		21	22	X	
	p	q	p	q	p	q	p	q	p	q	p	q	p	q	p	q	p	q	p	q	p	q	p	q	q	q	q	p	q	p	q	p	q	p	q	p	q	p	q	p	q
**Ren10473**	0	0	0	0	0	0	0	-1	0	0	0	0	0	0	0	0	-1	0	0	0	0	1	0	0	-1	0	0	1	0	0	0	1	0	2	0	0	0	1	1	0	0
**Ren10768**	0	0	1	1	0	0	0	0	0	0	0	0	1	0	0	1	0	0	0	0	0	0	0	0	-1	0	0	0	-1	0	0	0	NA	1	1	0	0	0	0	1	0
**Ren11122D**	0	0	0	0	-1	-1	0	0	0	0	0	-1	0	0	1	1	-1	-1	0	0	0	0	0	0	0	0	0	0	0	-1	1	0	0	0	0	0	0	-1	0	0	0
**Ren11122E**	0	0	0	1	-1	-1	0	0	0	0	0	0	0	0	0	0	-1	-1	-1	0	0	0	0	0	0	0	0	0	-1	-1	1	1	0	0	0	0	0	0	0	0	0
**Ren11122F**	0	0	0	0	-1	-1	0	0	0	0	0	**0**	1	0	0	0	-1	-1	-1	0	0	0	0	0	0	0	0	0	0	-1	1	0	0	0	0	0	0	0	0	0	0
**Ren11145C**	0	0	1	1	0	0	1	0	1	1	1	0	1	1	0	0	0	0	-1	0	0	0	0	0	-1	0	0	0	0	0	1	0	0	2	0	2	0	1	1	0	0
**Ren11175B**	0	0	0	0	0	0	0	0	0	0	0	0	0	0	0	0	0	0	0	1	1	1	1	1	-1	0	0	1	1	1	0	0	0	0	1	1	1	1	0	1	0
**Ren11175C**	0	0	0	0	0	0	0	0	0	0	0	0	0	0	0	0	0	-1	0	0	0	0	1	1	-1	0	0	1	0	0	0	0	-1	0	0	1	0	1	0	0	0
**Ren11175D**	0	0	0	0	0	0	0	0	0	0	0	0	0	0	0	0	-1	0	0	0	1	0	1	1	-1	0	0	0	0	0	0	0	0	0	1	0	0	0	0	0	0
**Ren11175F**	0	0	1	1	0	0	0	0	0	0	0	0	0	0	0	1	0	0	0	1	0	0	1	1	0	0	0	0	0	0	0	0	0	0	0	1	1	0	0	1	0
**Ren11175H**	0	0	1	1	0	0	0	0	0	1	1	0	0	0	0	0	0	0	0	0	1	1	0	0	-1	0	0	1	1	0	0	1	0	0	1	1	1	0	0	1	0
**Ren11175i**	0	0	0	0	0	0	1	1	0	1	0	0	0	0	0	0	0	0	0	0	1	1	1	1	0	0	0	0	0	0	1	0	0	0	1	0	0	0	0	1	0
**Ren11175J**	0	0	0	0	0	0	1	1	0	0	0	0	0	0	0	0	0	0	0	0	1	0	0	0	0	0	0	1	1	0	0	1	0	0	1	1	0	0	0	1	0
**Ren11175K**	0	0	0	0	0	0	0	0	1	1	0	0	0	0	0	0	0	0	0	1	1	0	1	0	0	0	0	0	0	1	0	0	0	0	0	0	0	1	0	1	0
**Ren11201**	0	0	1	0	0	0	0	0	0	0	0	0	0	0	0	0	0	0	0	0	0	1	0	0	-1	1	0	1	0	0	0	0	0	1	1	1	0	0	1	0	0
**Ren11244**	0	0	0	0	0	0	0	-1	0	0	0	0	0	0	0	0	-1	-1	0	0	0	0	0	0	0	0	0	0	0	-1	0	0	0	0	0	0	0	0	0	0	0
**Ren11253**	0	0	0	0	0	0	0	0	0	0	0	0	0	0	1	1	-1	-1	0	0	0	0	0	0	0	0	0	1	0	0	1	0	0	0	0	0	0	0	0	0	0
**Ren11324D**	0	0	0	0	0	0	-1	-1	1	1	0	0	0	0	-1	0	-1	-1	0	0	0	0	0	0	-1	0	0	0	0	0	0	0	-1	0	0	0	0	0	0	0	0
**Ren11325H**	0	0	1	0	0	0	1	0	0	1	0	1	0	0	0	1	0	0	0	0	1	0	0	1	0	0	0	0	0	0	0	1	0	0	0	0	0	1	0	0	0
**Ren11535**	0	0	0	0	0	0	0	0	0	0	1	0	0	1	-1	0	0	-1	-1	0	1	1	0	1	0	0	0	1	0	0	0	0	-1	0	0	0	0	0	1	0	0
**Ren11619B**	0	0	0	0	0	0	0	0	0	0	0	0	0	1	0	0	1	0	-1	0	1	1	0	0	-1	0	0	1	0	0	0	0	0	0	0	0	0	0	1	0	0
**Ren11644**	0	0	0	0	-1	0	0	0	0	0	0	0	1	1	0	1	-1	-1	0	0	0	0	0	1	0	-1	0	0	0	0	0	0	0	0	0	0	0	0	0	0	0
**Ren11670**	0	0	1	1	0	0	0	0	0	0	1	1	1	1	-1	0	0	0	0	1	1	0	0	0	0	0	0	1	1	1	0	0	0	1	1	1	0	1	0	1	0
**Ren11845**	1	1	1	1	0	0	0	0	0	0	1	0	1	0	-1	0	0	0	0	1	0	0	0	0	0	0	0	1	1	1	0	0	NA	1	1	0	0	1	0	0	0
**Ren11965**	-1	1	0	0	-1	0	0	-1	1	1	0	0	0	0	-1	0	-1	-1	0	0	0	0	0	0	0	0	0	0	0	0	0	0	0	0	0	0	0	0	0	0	0
**Ren12147**	0	0	1	1	0	0	0	0	0	0	1	0	0	0	0	0	0	0	0	0	1	1	0	0	0	1	0	0	0	0	0	0	0	0	1	0	0	0	0	0	0
**Ren12296**	0	0	0	0	0	0	0	0	0	0	0	-1	1	1	-1	0	0	0	0	0	1	0	1	1	-1	0	1	1	0	0	0	-1	NA	0	0	0	0	0	0	0	0
**Ren12449**	0	0	0	0	0	0	0	0	0	0	0	0	0	0	1	0	0	-1	0	0	1	1	0	0	0	0	0	1	0	-1	0	0	0	0	0	1	0	0	1	1	0

Table S2: Prediction of CNVs from RNAseq

red – gain of 1 (light red) or 2 (dark red) copies of chromosome arm; 0/white – diploid chromosome arm; -1/blue – loss of chromosome arm; NA/grey – no information
